# *Fusobacterium nucleatum* tumor DNA levels are associated with survival in colorectal cancer patients

**DOI:** 10.1007/s10096-019-03649-1

**Published:** 2019-07-31

**Authors:** Andrew T. Kunzmann, Marcela Alcântara Proença, Haydee WT Jordao, Katerina Jiraskova, Michaela Schneiderova, Miroslav Levy, Václav Liska, Tomas Buchler, Ludmila Vodickova, Veronika Vymetalkova, Ana Elizabete Silva, Pavel Vodicka, David J. Hughes

**Affiliations:** 1 Centre for Public Health, Queen’s University Belfast, Belfast, Northern Ireland; 2 Department of Biology, São Paulo State University, UNESP, São José do Rio Preto, SP Brazil; 3 Institute of Biology and Medical Genetics, First Faculty of Medicine, Charles University, Prague, Czech Republic; 4 Department of Molecular Biology of Cancer, Institute of Experimental Medicine of the Czech Academy of Sciences, Prague, Czech Republic; 5 Department of Surgery, General University Hospital in Prague, Prague, Czech Republic; 6 Department of Surgery, First Faculty of Medicine, Charles University and Thomayer Hospital, Prague, Czech Republic; 7 Biomedical Centre, Faculty of Medicine in Pilsen, Charles University, Pilsen, Czech Republic; 8 Department of Oncology, First Faculty of Medicine, Charles University and Thomayer Hospital, Prague, Czech Republic; 9 Cancer Biology and Therapeutics Group, School of Biomolecular and Biomedical Science, UCD Conway Institute, University College Dublin, Dublin, D04 V1W8 Ireland

**Keywords:** Colorectal neoplasm, Colorectal cancer, *Fusobacterium nucleatum*, Bacterial infection, Disease survival

## Abstract

There is increasing evidence indicating a role for *Fusobacterium nucleatum* (*F. nucleatum*) in colorectal cancer (CRC) development and prognosis. This study evaluated *F. nucleatum* as a prognostic biomarker, by assessing its association with post-diagnosis survival from CRC. From September 2008 to April 2012 CRC patients (*n* = 190) were recruited from three hospitals within the Czech Republic. *F. nucleatum* DNA copies were measured in adjacent non-malignant and colorectal tumor tissues using quantitative real-time PCR. Cox Proportional Hazards (HR) models were applied to evaluate the association between *F. nucleatum* DNA and overall survival, adjusting for key confounders. Risk prediction modeling was conducted to evaluate the ability to predict survival based on *F. nucleatum* status. High, compared with low, levels of *F. nucleatum* in colorectal tumor tissues were associated with poorer overall survival (adjusted HR 1.68, 95% CI 1.02–2.77), which was slightly attenuated after additional adjustment for microsatellite instability status. However, inclusion of *F. nucleatum* in risk prediction models did not improve the ability to identify patients who died beyond known prognostic factors such as disease pathology staging. Although the increased presence of *F. nucleatum* was associated with poorer prognosis in CRC patients, this may have limited clinical relevance as a prognostic biomarker.

**Electronic supplementary material:**

The online version of this article (10.1007/s10096-019-03649-1) contains supplementary material, which is available to authorized users.

## Introduction

Accumulating evidence indicates a potential association of microbiome dysbiosis with colorectal cancer (CRC) development and prognosis [1, 2].

*Fusobacterium nucleatum* (*F. nucleatum*) is an anaerobic, gram-negative commensal pathogen that is associated with several human diseases, especially those related to the oral and intestinal tract [3, 4]. Numerous studies have shown higher abundance of *F. nucleatum* DNA or RNA in CRC tumors versus surrounding non-malignant mucosa, or in stool sample DNA from CRC patients compared to controls [5–12] (reviewed in [13]). These findings are consistent with in vitro and animal model evidence and human studies indicating an etiological role of *F. nucleatum* in colorectal carcinogenesis through promotion of an immunocompromised proinflammatory microenvironment favorable to tumor initiation and progression [5, 14–17]. Alternatively, growth of *F. nucleatum* may simply reflect an opportunistic invader capable of surviving the harsh hypoxic conditions of developing tumors [8].

Regarding CRC pathology and clinical characteristics, high levels of *F. nucleatum* in stool and tumor tissue have been associated with right-sided tumors, higher stage, molecular subtypes, and worse clinical outcome (reviewed in [7, 18–20]). Several studies in diverse geographical settings, following the initial observation by Flanagan et al. (2014) [11], suggest that *F. nucleatum* may affect clinical outcome for CRC patients. These studies observed that higher tumor *F. nucleatum* levels were associated with poorer overall survival (OS) in CRC patients from the Czech Republic, China, Japan [11, 21–23], and metastatic CRC in South Korea [24]. The Chinese study from Wei et al. also reported an association with lower disease-free survival (DFS) [22]. Although a US study reported no association between high tumor *F. nucleatum* levels and OS in a large cohort of CRC patients, they did observe an association with shorter CRC-specific survival independent of clinical, pathological, and tumor molecular characteristics [25].

However, given the limited number of published studies to date, there is a need to further examine the relationship between *F. nucleatum* and CRC prognosis, and to what extent measurement of *F. nucleatum* DNA may serve as a prognostic biomarker. The aims of this study were to assess the association between *F. nucleatum* DNA levels from tumor and adjacent non-malignant tissue with survival outcomes in a much larger and separate cohort of 190 Czech patients with CRC to our initial study suggesting a link with poorer overall patient survival [11].

## Materials and methods

### Study population

This cohort study included 190 adult patients diagnosed with histologically confirmed CRC. All subjects were recruited from September 2008 to April 2012 from one of three hospitals in the Czech Republic (General University Hospital, Thomayer Hospital in Prague and Biomedical Centre, Faculty of Medicine in Pilsen). Table 1 lists the participant characteristics.

**Table 1:** Participant characteristics in a cohort of 190 colorectal cancer patients based on *F. nucleatum* DNA status in colorectal cancer tissue

	Low tumor *F. nucleatum*^1^		High tumor *F. nucleatum*^2^		*P* value
	No.	%	No.	%	
Total	129	(68.2)	61	(31.8)	
Age at surgery					
Under 60	22	(17.1)	9	(14.8)	0.95
60–< 70	51	(39.5)	23	(37.7)	
70–< 80	36	(27.9)	19	(31.1)	
80+	20	(15.5)	10	(16.4)	
Sex					
Female	40	(31)	20	(32.8)	0.81
Male	89	(69)	41	(67.2)	
Tumor stage					
I	25	(19.4)	9	(14.8)	0.238
II	36	(27.9)	23	(37.7)	
III	44	(34.1)	14	(23)	
IV	22	(17.1)	14	(23)	
*Unknown*	2	(1.6)	1	(1.6)	
Tumor location					
Proximal colon	39	(30.2)	22	(36.1)	0.58
Distal colon	38	(29.5)	14	(23)	
Rectal	52	(40.3)	25	(41)	
Neo/adjuvant treatment^3^					
No	89	(69)	34	(55.7)	0.07
Yes	40	(31)	27	(44.3)	
Smoking history					
Never	48	(43.6)	24	(45.3)	0.84
Ever	62	(56.4)	29	(54.7)	
*Missing*	19	(14.5)	8	(13.1)	
Body Mass Index					
18.5–< 25	49	(38)	18	(29.5)	0.61
25–< 30	39	(30.2)	21	(34.4)	
30+	16	(12.4)	7	(11.5)	
*Missing*	25	(19.4)	15	(24.6)	
*F. nucleatum* DNA in non-malignant, adjacent tissue					
Low^1^	117	(90.7)	36	(59.0)	
High^2^	10	(7.8)	24	(39.3)	< 0.001
*Missing*	2	(1.6)	1	(1.6)	
Microsatellite instability status					
MSS/MSI-L	105	(81.4)	40	(65.6)	
MSI-H	3	(2.3)	10	(16.4)	< 0.001
*Missing*	21	(16.3)	11	(18.0)	
*KRAS* status					
Wild type	57	(44.2)	18	(29.5)	
Mutated	30	(23.3)	17	(27.9)	0.15
*Missing*	42	(32.6)	26	(42.6)	
*NRAS* status					
Wild type	80	(62.0)	34	(55.7)	
Mutated	7	(5.4)	1	(1.6)	0.30
*Missing*	42	(32.6)	26	(42.6)	
*BRAF* status					
Wild type	81	(62.8)	31	(50.8)	
Mutated	6	(4.7)	4	(6.6)	0.41
*Missing*	42	(32.6)	26	(42.6)	
*PIK3CA* status					
Wild type	78	(60.5)	31	(50.8)	
Mutated	9	(7.0)	4	(6.6)	0.86
*Missing*	42	(32.6)	26	(42.6)	

^1^No *F. nucleatum* DNA detected within 42 PCR cycles or *F. nucleatum* (2^−∆CT^) below 0.0008 (i.e., the median among individuals with tumor *F. nucleatum* DNA detected in 42 PCR cycles)

^2^*F. nucleatum* (2^−∆CT^) above 0.0008

^3^Neo-adjuvant or adjuvant radiotherapy or chemotherapy within 6 months of surgery

### Data collection and follow-up

A structured questionnaire assessed patients’ personal characteristics (either self-reported or through interviews with their doctors), including date of birth, sex, lifestyle habits, body mass index (BMI), diabetes, and personal/family history of cancer. Clinicians recorded information about tumor location, tumor-node-metastasis (TNM) stage system, degree of tumor differentiation, and adjuvant chemotherapy treatment details. Patients with Lynch syndrome, polyposis syndromes, inflammatory bowel disease, or incomplete information on baseline characteristics were excluded. Follow-up information about distant metastasis, relapse, and date of death was also recorded and all registered patients were followed from surgery to death or to the end of the study (September 2018).

### DNA extraction

After surgical resection, colorectal tumor and adjacent non-malignant tissue samples were freshly frozen and conserved in either liquid nitrogen or RNA later and stored at − 80 °C. DNA was extracted from colorectal and adjacent tissues using the DNeasy Blood and Tissue Kit and by following the manufacturer’s instructions (Qiagen, Courtaboeuf, France). The DNA concentration was determined using the Quant-IT dsDNA BR Assay Kit (Life Technologies Czech Republic s.r.o., Prague, Czech Republic) and an Infinite M200 fluorescence reader (Tecan Group Ltd., Männedorf, Austria). Microsatellite instability (MSI) status was determined in tumor tissue and non-malignant adjacent mucosa using a pentaplex PCR assay of 5 mononucleotide repeat markers (BAT25, BAT26, NR21, NR24, NR27) with fluorescently labeled primers and standard PCR chemistry [26]. Fragment analysis was performer on ABI 3130 (Applied Biosystems, Foster City, CA, USA). The final comparison between tumor and non-tumor DNA short tandem repeat profiles was performed with GeneMapper v4.1 software (Applied Biosystems, Foster City, CA, USA). A tumor specimen was classified as MSI-high when 2 or more loci were unstable [26]. Mutation analysis for *BRAF*, *KRAS*, *NRAS*, and *PIK3CA* was performed using a Randox mutation biochip array (Randox Laboratories Ltd., Crumlin, Northern Ireland) according to the manufacturer’s instructions, as previously described [27].

### Quantitative real-time polymerase chain reaction

Quantitative real-time polymerase chain reaction (qPCR) measured the number of copies of *F. nucleatum* DNA (*nusG* gene), and a control human prostaglandin transporter (*PGT*) gene, in the colorectal tumor and non-malignant adjacent tissue using an Applied Biosystems 7500 Real-Time PCR System Levels, as previously detailed [11]. qPCR assays for *F. nucleatum* were performed in duplicate for each sample, and a cutoff level of 42 replication cycles (cycle threshold; CT) for both duplicate reactions was used to determine presence of *F. nucleatum* DNA. Bacterial abundance was calculated in both colorectal tumor tissue and adjacent tissue by 2^−∆CT^, where ∆CT is the difference in the CT number for *F. nucleatum* and the *PGT* reference gene assay. *F. nucleatum* abundance (in individuals with the bacterium detected) was divided into low and high groups based on values below or above the median value, respectively.

### Statistical analysis

All statistical analyses were performed using Stata version 14 (StataCorp, College Station, TX, USA).

The characteristics of patients based on the prevalence of *F. nucleatum* were compared using chi-squared tests. Kaplan-Meier curves and Cox Proportional Hazards ratio (HR) models with 95% confidence intervals (95% CI) were applied to evaluate the association between *F. nucleatum* in colorectal tissue and overall survival. Both univariate and multivariate analyses were conducted, adjusted for important confounders such as age, sex, tumor stage, and chemotherapy or radiotherapy.

Sensitivity analyses assessed whether the association remained when conducting complete case analysis and separately when excluding deaths within the first 6 months. Further models included additional potential confounders associated with a 10% change in coefficient between *F. nucleatum* and OS, when added individually to the main multivariate model. The factors tested for inclusion were molecular characteristics (MSI status, mutations for *BRAF*, *KRAS*, *NRAS*, and *PIK3CA*) and lifestyle factors (including BMI and smoking history), tumor location, and the presence of *F. nucleatum* in adjacent non-malignant tissue. MSI status and *F. nucleatum* in adjacent non-malignant tissue were selected as additional covariates. Continuous and tertile category analyses were also conducted for assessing both *F. nucleatum* tumor abundance (2^−∆CT^) and bacterial quantification in disease tissue over adjacent-matched colorectal tissue (2^−∆∆CT^). The three categories included “no/low,” where values of *F. nucleatum* were not detectable or if the quantification was in the lowest tertile group; “moderate” if bacterial abundance was in the middle tertile category; or “high” if *F. nucleatum* quantification was in the highest tertile rank.

In subgroup analyses, likelihood ratio tests assessed whether the association differed by age, sex, tumor stage, tumor location, chemotherapy or radiotherapy, smoking history, BMI, or by molecular characteristics (including status of MSI, *BRAF*, *KRAS*, *NRAS*, and *PIK3CA*).

The use of *F. nucleatum* load as a prognostic marker was examined by assessing the area under the receiver operating characteristics (ROC) curve of existing prognostic markers (tumor stage, age, sex, cancer treatment, and MSI status) when including and excluding tumor *F. nucleatum* status (high versus low).

## Results

This study included 190 CRC patients who were followed up for a median of 2.34 years (range 0.01–6.77), during which time 71 patients died.

As observed in several studies [13], *F. nucleatum* was significantly more abundant in the tumor tissue compared to the matched surrounding mucosa (*p* = 0.002). Table 1 outlines the participant characteristics according to the abundance of *F. nucleatum* in CRC tumor tissue. One third of CRC patients had high *F. nucleatum* abundance in their tumor tissue. Patients with low and high abundance of *F. nucleatum* were similar in terms of age, sex, tumor stage and location, neo-adjuvant treatment receipt, smoking history, and BMI. Patients with high tumor *F. nucleatum* were more likely to have MSI tumors than patients with low tumor *F. nucleatum* and tended to have a higher abundance of *F. nucleatum* in the adjacent-matched non-malignant tissue.

**Fig. 1:**
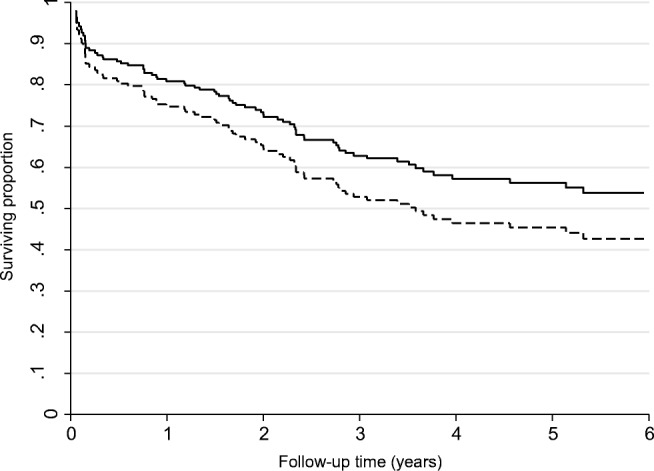
Kaplan-Meier curve showing the overall survival of colorectal cancer patients by the amount of *F. nucleatum* DNA in colorectal tumor tissue (2^−∆CT^); continuous line indicates low tumor *F. nucleatum*; broken line indicates high tumor *F. nucleatum*. In the adjusted models, high *F. nucleatum* was associated with poorer OS compared to lower levels (HR 1.68, 95% CI 1.02–2.77, *p* = 0.04)

The association between tumor *F. nucleatum* status and overall survival is displayed in Table 2 and Fig. 1. There was no association with survival in the unadjusted analyses. However, high *F. nucleatum* was associated with poorer OS compared to low levels of the bacterium in colorectal tissue in fully adjusted models (HR 1.68, 95% CI 1.02–2.77, *p* = 0.04). Further adjustment for MSI status and *F. nucleatum* abundance in adjacent normal tissue did not markedly change this result, although it did lose statistical significance (HR 1.80, 95% CI 0.97–3.28, *p* = 0.06; see Supplementary Table 1). Additional factors considered in the adjustment model, comprising tumor colorectal subsite location, smoking history, BMI, and mutations of *KRAS*, *NRAS*, *BRAF*, and *PIK3CA*, did not meet the 10% change in coefficient criteria for confounder selection.

**Table 2:** Cox Proportional Hazards models for the association between *Fusobacterium nucleatum* DNA in colorectal cancer tissue and overall survival

*F. nucleatum* DNA in colorectal cancer tissue (2^−∆CT^)	Person-years	No. of events	Unadjusted		Adjusted^1^	
			HR (95% CI)	*p* value	HR (95% CI)	*p* value
Low/no	376.2	44	1.00 (referent)	–	1.00 (referent)	–
High	156.0	27	1.37 (0.85–2.22)	0.20	1.68 (1.02–2.77)	0.04

^1^Adjusted for age (< 60, 60–< 70, 70–< 80, 80+), sex (men, women), tumor stage (I, II, III, IV, missing), and chemotherapy or radiotherapy within 6 months (no, yes)

Sensitivity analyses for whether the association remained when conducting complete case analysis or after excluding deaths within the first 6 months did not substantially change the HR estimates, although the latter lost significance (HR 1.71, 95% CI 0.94–3.10; Supplementary Table 1). No associations between tumor *F. nucleatum* status and OS were observed when analyses were stratified by CRC molecular subtypes (including *BRAF*, *KRAS*, *NRAS*, and *PIK3CA* mutation status) (Supplementary Table 1). The association between *F. nucleatum* and survival was significant in patients aged over 70 years old (HR 2.23, 95% CI 1.15–4.35, *p* = 0.02), in patients with left-sided tumors (HR 2.34, 95% CI 1.25–4.37, *p* = 0.008), and in those patients not receiving chemotherapy and/or radiotherapy (HR 1.87, 95% CI 1.02–3.45, *p* = 0.04). However, formal tests for statistical interaction were not significant. Other clinical factors such as smoking status did not significantly modify the associations observed between *F. nucleatum* and OS (Supplementary Table 1).

Considering bacterial presence in the surrounding non-malignant mucosal tissue, a multivariate analysis using relative quantification of *F. nucleatum* (from the mucosal and tumor levels) showed no association with OS (HR 1.04, 95% CI 0.62–1.76; Supplementary Table 1). However, tumor levels adjusted for the corresponding *F. nucleatum* levels in the surrounding mucosal tissue (and the main confounders) were associated with poorer OS (HR 1.94, 95% CI 1.09–3.46; Supplementary Table 1).

Assessing *F. nucleatum* levels in a continuous model provided null results (HR 1.00, 95% CI 0.99–1.00). We were underpowered to conduct a restricted cubic spline analysis to explore the curve for the continuous model. However, although they were not significant, categorical analyses of no/low, moderate, or high bacterial levels interestingly suggest that this is largely due to a non-linear relationship where CRC cases with moderate *F. nucleatum* have possibly better or equivalent survival than those with no/low *F. nucleatum* in their tumor tissues (see Supplementary Table 2). Survival then decreases with higher bacterial load, as also indicated by the Kaplan-Meier survival curves in our prior report from 2014 (see figure 3, Flanagan et al., 2014 [11]). Alternatively, this may simply reflect lower power for these tertile groupings. Repeating these categorical analyses by the delta-delta Ct method used in Flanagan et al. [11] for comparing differences in bacterial levels between tumor and matched mucosal tissues, or by adjusting for *F. nucleatum* mucosal tissue levels, made no major differences to the results (Supplementary Table 2).

Finally, when the utility of *F. nucleatum* as a prognostic marker was assessed by ROC curves, there was no improvement in the area under the curve when added to traditional prognostic markers or data such as tumor stage, age, sex, treatment, and MSI status (Table 3).

**Table 3:** Performance of prognostic models for five-year overall survival in colorectal cancer patients when including and excluding tumor *F. nucleatum* status

	AUROC (95% CI)
Tumor stage	0.68 (0.60–0.76)
Tumor stage + *F. nucleatum*	0.69 (0.61–0.77)
Age, sex, tumor stage	0.78 (0.72–0.85)
Age, sex, tumor stage + *F. nucleatum*	0.78 (0.71–0.85)
Age, sex, tumor stage, treatment	0.78 (0.71–0.84)
Age, sex, tumor stage, treatment + *F. nucleatum*	0.78 (0.71–0.84)
Age, sex, tumor stage, treatment, and MSI status	0.83 (0.77–0.89)
Age, sex, tumor stage, treatment, and MSI status + *F. nucleatum*	0.83 (0.77–0.89)

*F. nucleatum*, *Fusobacterium nucleatum*; *AUROC*, area under receiver operating characteristic curve, 95% *CI*, 95% confidence intervals; *MSI*, microsatellite instability

## Discussion

In this study, we found that high *F. nucleatum* DNA in colorectal tumor tissue is associated with shorter survival in a cohort of CRC patients from the Czech Republic. However, prognostic modeling revealed that *F. nucleatum* may not lead to notable improvements in our ability to predict patient prognosis beyond other known indicators, such as tumor stage.

The association of higher levels of *F. nucleatum* DNA with a shorter survival in CRC patients is concordant with previous reports [11, 21–25]. These include our initial study suggesting this link for the first time, although in a small, separate group of 32 Czech CRC patients giving imprecise HR estimates with wide confidence intervals [11]. The large study by Mima et al., of tissue from 1069 CRC cases within the Nurses’ Health Study and the Health Professionals Follow-Up study, found that high *F. nucleatum* was more strongly associated with CRC-specific survival (HR 1.58, 95% CI 1.04–2.39) than with OS (HR 1.08, 95% CI 0.76–1.52) [25]. Given the long duration of follow-up in this study (median of 10.7 years), analyses of OS understandably incorporated a high proportion of non-CRC deaths (46%), likely underlying the null association with OS for *F. nucleatum*. Considering the other two of these reports which calculated HR values, then the strength of the association for higher *F. nucleatum* with worse OS seen in the current study (HR 1.68, 95% CI 1.02–2.77) is similar to Chinese CRC patients (multivariate HR 1.99, 95% CI 1.02–3.88) [22] and very close to the estimate for a South Korean metastatic CRC cohort (multivariate HR 1.69, 95% CI 1.04–2.75) [24]. Therefore, the current study provides additional support that *F. nucleatum* is independently associated with poorer OS for CRC patients. Contrastingly, a recent and separate South Korean study reported no association of bacterial levels with survival for 593 stage II (high-risk) and stage III CRC patients whom had all received oxaliplatin-based adjuvant chemotherapy [28]. This aligns with our observation of no association between *F. nucleatum* and OS in patients who had received chemotherapy or radiotherapy. Furthermore, in subgroup analyses, these authors also reported that higher *F. nucleatum* showed a non-significant tendency toward worse prognosis in sigmoid colon and rectal cancer but was significantly associated with a more favorable DFS for non-sigmoid colon cancer patients that were also non-MSI-high (i.e., MSS/MSI-low). We observed that the association of higher bacterial levels with worse prognosis was more apparent in patients with left-sided tumors (thus including sigmoid and rectal cancers, as well as the splenic flexure and descending colon). However, this Korean study was likely seriously confounded by study design and methodology issues such as the lack of qPCR data on adjacent-matched tissues for each tumor, and that FFPE tissue-derived DNA was used to estimate the bacterial load resulting in a high number of failed PCRs for the *PGT* control (21%) so that the results are possibly unreliable. Indeed, tissue fixation method and storage time has been shown to affect *F. nucleatum* qPCR positivity [24, 28].

The biological mechanism for the association between *F. nucleatum* and patient survival has not been fully elucidated. The bacterium has been shown to persist in cancer cells from metastatic CRC lesions and *Fusobacterium*–culture positive tumors resulted in more successful xenografts in mice, suggesting an association with more severe disease [29]. Human and functional studies provide evidence that *F. nucleatum*–mediated increased gut inflammation and chemoresistance, through immune signaling and autophagy activation, largely explains the poorer prognosis for CRC patients [22, 30]. *F. nucleatum* may invade into tumor cells and exacerbate inflammatory and oncogenic responses via FadA adhesion to E-cadherin with beta-catenin pathway activation [5] and through Fap2 binding of the Gal-GalNAc polysaccharide overexpressed by CRC cells [15]. Additionally, Fap2 interaction with the TIGIT receptor expressed on natural killer cells may enhance immune evasion [17]. The bacterium is also associated with upregulation of several inflammatory cytokines through a postulated miRNA-mediated activation of *TLR2/TLR4* [31] and activation of the JAK/STAT and MAPK/ERK pathways linked to CRC tumor progression [32]. Alternatively, as in the case for any role in initial colorectal carcinogenesis, *F. nucleatum* may not directly affect CRC disease severity, but simply thrive in the increasingly hostile tumor microenvironment of aggressive tumors that are likely to be associated with a poorer prognosis. In contrast to the suggested role of chemoresistance, in subgroup analyses, we only observed an association between higher *F. nucleatum* and OS in patients who did not receive chemotherapy or radiotherapy. However, our power was very limited to adequately evaluate this due to low numbers of patients with both high bacterial levels and relevant therapy data, and formal tests for statistical interaction were not significant. Further analyses by continuous or category (no/low, moderate, and high) levels of *F. nucleatum* possibly suggest that moderate amounts may be conversely associated with better or equal survival than for patients with no/low presence of the bacterium in their tumor tissue. Conceivably, this could be through triggering of an increased immune response that may combat tumor progression/recurrence until the bacterium reaches higher amounts that would compromise therapy and/or make the cancer more aggressive. Alternatively, this may again reflect a non-causative correlation between higher bacterial levels in more severe cancers. Although, considerable caution is required in assessing these results, as none were significant and there was lower power for these evaluations.

In analyses by CRC molecular subtypes, we found that the association between *F. nucleatum* and overall survival was no longer statistically significant after additional adjustment for MSI status and presence of the bacterium in adjacent non-malignant tissue. This may be attributable to low statistical power, as few individuals were MSI-high (*n* = 13). Stratified analysis in patients with MSI-low/stable colorectal tumors, or by *BRAF*, *KRAS*, *NRAS*, and *PIK3CA* mutation status, also showed no evidence of an association between *F. nucleatum* and OS. Unfortunately, we had insufficient statistical power to conduct this analysis for MSI-high tumors. Nonetheless, a strong association between the amount of *F. nucleatum* in CRC tissue and MSI-high was observed in the study by Mima et al. [25]. Although MSI-high status is generally perceived as a favorable prognostic indicator, a large cohort study of Stage II/III colon cancer patients from Northern Ireland defined a subgroup of patients having MSI-high tumors with a high fibroinflammatory score that were associated with a 2.5-fold increased risk of death [33]. The fibroinflammatory score is indicative of tumor microenvironment features such as peritumoral diffuse lymphoid inflammation, Crohn’s disease-like reaction, and tumor stromal proportions [33]. However, as we were unable to measure this directly with our limited sample size, it would be interesting for further studies to test whether such a group of MSI-high/fibroinflammatory-high tumors correlate with increased *F. nucleatum* abundance and poorer outcomes. Additionally, a positive and negative correlation has been reported for MSS/MSI-low and MSI-high CRCs, respectively, with higher *F. nucleatum* levels and a clinically favorable increased tumor-infiltrating lymphocyte (TIL) density [34]. Thus, the association of increased TILs with *F. nucleatum* load in non-MSI-high tumors may improve the disease outcomes for these patients [28]. Overall, it appears that high *F. nucleatum* abundance is contributing to an adverse inflammatory tumor microenvironment that is associated with poorer outcomes, although tumor location and molecular subtype may be important factors modifying the influence of *F. nucleatum* on patient outcomes.

Additionally, *F. nucleatum* did not appear to be a useful prognostic tool for predicting individuals who may have worse survival when added to current known prognostic factors such as tumor stage. Although, we did not have information on CRC-specific survival, which may be more strongly related to *F. nucleatum*, it is likely that most deaths in this cohort were attributed to CRC considering the relatively short follow-up time (median of 2.34 years). Further, larger cohort studies with information on causes of death could examine the utility of *F. nucleatum* measurement regarding CRC-specific survival prognosis, with adequately powered analyses to assess whether the prognostic role is greater in certain tumor subsites, molecular subtypes, or patient subgroups.

Our study had several notable strengths and limitations. Strengths include the use of DNA extracted from fresh-frozen tissue, with no tissue fixation, so that we had > 99% successful qPCR assays (based on consistent amplification of the human *PGT* reference gene in all tumor and matched mucosa samples), and that we assessed the prognostic utility of *F. nucleatum* DNA measurement in CRC patients. However, the survival analyses were constricted to OS so we were not able to assess CRC-specific mortality, the cohort size was limited for many of the subgroup analyses, and that qPCR may not measure *F. nucleatum* as accurately as other approaches, such as droplet digital PCR used by Yamaoka et al. [23]. However, qPCR is the most widely accepted and used method of *F. nucleatum* measurement in other studies.

In conclusion, our study adds to the observational evidence of an association between high abundance of *F. nucleatum* in colorectal tumors and poorer survival outcomes. Additionally, as antibiotic treatment of colon cancer xenografts in mice has been shown to decrease *F. nucleatum* levels and tumor growth [29], trials testing whether treatments aimed at reducing the tumor *F. nucleatum* lead to improved clinical outcomes for CRC patients may be warranted. Although, our study further indicated that the utility of *F. nucleatum* for predicting prognosis is limited, this needs to be further assessed in independent datasets.

## Electronic supplementary material

ESM 1 (DOCX 20 kb)

ESM 2 (DOCX 16 kb)
