# Supplementary material for: Fusobacterium nucleatum tumor DNA levels are associated with survival in colorectal cancer patients
Source: Eur J Clin Microbiol Infect Dis. 2019 Jul 31;38(10):1891–9. doi: 10.1007/s10096-019-03649-1 (PMC6778531; doi:10.1007/s10096-019-03649-1)
Supplement: Supplementary file 1 — (DOCX 20 kb) [file 10096_2019_3649_MOESM1_ESM.docx]

**Supplementary Table 1. Sensitivity analyses using Cox Proportional Hazards models for the association between *Fusobacterium nucleatum* DNA status and overall survival in multivariate analyses.**

|  |  |  | Low tumor  *F. Nucleatum* |  |  | High tumor  *F. Nucleatum* |  |
| --- | --- | --- | --- | --- | --- | --- | --- |
|  | Person-years | No. of events | HR (95% CI)^1^ | Person-years | No. of events | HR (95% CI)^1^ | *P*-int |
| Main analysis | 376.2 | 44 | 1.00 (referent) | 156.0 | 27 | 1.68 (1.02-2.77) | - |
| Additional adjustments^2^ | 376.2 | 44 | 1.00 (referent) | 156.0 | 27 | 1.80 (0.97-3.28) | - |
| Complete case analysis | 366.0 | 43 | 1.00 (referent) | 156.7 | 27 | 1.70 (1.03-2.80) | - |
| Exclude deaths within 6 months | 318.4 | 34 | 1.00 (referent) | 133.6 | 18 | 1.71 (0.94-3.10) | - |
| Age |  |  |  |  |  |  |  |
| <70 years | 212.9 | 20 | 1.00 (referent) | 106.0 | 7 | 0.87 (0.34-2.24) | 0.07 |
| 70+ years | 163.3 | 24 | 1.00 (referent) | 53.9 | 20 | 2.23 (1.15-4.35) |  |
| Sex |  |  |  |  |  |  |  |
| Women | 137.4 | 9 | 1.00 (referent) | 57.0 | 6 | 3.57 (0.96-13.25) | 0.60 |
| Men | 238.8 | 35 | 1.00 (referent) | 103.0 | 21 | 1.42 (0.79-2.56) |  |
| Tumor stage |  |  |  |  |  |  |  |
| I-III | 325.9 | 30 | 1.00 (referent) | 146.5 | 17 | 1.49 (0.80-2.79) | 0.88 |
| IV | 40.1 | 13 | 1.00 (referent) | 10.2 | 10 | 2.67 (0.91-7.85) |  |
| Tumor location |  |  |  |  |  |  |  |
| Left-sided | 259.4 | 29 | 1.00 (referent) | 90.1 | 20 | 2.34 (1.25-4.37) | 0.50 |
| Right-sided | 116.8 | 15 | 1.00 (referent) | 69.8 | 7 | 0.97 (0.34-2.75) |  |
| Chemotherapy/  Radiotherapy |  |  |  |  |  |  |  |
| No | 253.1 | 31 | 1.00 (referent) | 77.6 | 19 | 1.87 (1.02-3.45) | 0.35 |
| Yes | 123.1 | 13 | 1.00 (referent) | 82.4 | 8 | 1.07 (0.36-3.22) |  |
| Smoking history |  |  |  |  |  |  |  |
| Never smokers | 165.2 | 15 | 1.00 (referent) | 61.3 | 10 | 1.46 (0.61-3.49) | 0.41 |
| Ever smokers | 182.6 | 29 | 1.00 (referent) | 88.2 | 16 | 1.77 (0.89-3.53) |  |
| Body mass index (BMI; kg/m^2^) |  |  |  |  |  |  |  |
| <25 | 155.5 | 24 | 1.00 (referent) | 52.3 | 9 | 2.62 (0.86-8.03) | 0.79 |
| 25+ | 170.9 | 20 | 1.00 (referent) | 80.1 | 15 | 1.38 (0.65-2.92) |  |
| Microsatellite instability status |  |  |  |  |  |  |  |
| MSS/MSI-L | 288.4 | 32 | 1.00 (referent) | 92.0 | 13 | 1.35 (0.60-3.02) | - |
| *KRAS* status |  |  |  |  |  |  |  |
| Wild type | 161.5 | 57 | 1.00 (referent) | 58.6 | 18 | 1.28 (0.49-3.33) | 0.46 |
| Mutated | 93.4 | 30 | 1.00 (referent) | 33.5 | 17 | 2.74 (0.88-8.52) |  |
| *NRAS* status |  |  |  |  |  |  |  |
| Wild type | 241.5 | 80 | 1.00 (referent) | 89.0 | 34 | 1.79 (0.92-3.49) | - |
| Mutated | 13.4 | 7 | 1.00 (referent) | 3.16 | 1 | Not estimable |  |
| *BRAF* status |  |  |  |  |  |  |  |
| Wild type | 242.6 | 81 | 1.00 (referent) | 82.2 | 31 | 1.62 (0.81-3.26) |  |
| Mutated | 12.38 | 6 | 1.00 (referent) | 10.0 | 4 | Not estimable | - |
| *PIK3CA* status |  |  |  |  |  |  |  |
| Wild type | 224.6 | 78 | 1.00 (referent) | 77.5 | 31 | 1.50 (0.75-3.00) | - |
| Mutated | 30.4 | 9 | 1.00 (referent) | 14.7 | 4 | Not estimable |  |

^1^ All analyses adjusted for age (<60, 60-<70, 70-<80, 80+), sex (women, men), tumor stage (I, II, III, IV), and chemotherapy/radiotherapy within 6 months (no, yes), excluding stratifying variable.

^2^ Additionally adjusted for MSI status (MSS/MSI-L, MSI-H, missing) and *F. nucleatum* DNA in adjacent matched non-malignant tissue (Low/No, high, missing). Other factors including tumor location (Proximal colon, Distal colon, Rectal), smoking history (Never, ever, missing), body mass index (18.5-<25, 25-<30, 30+, Missing), and mutations of *KRAS*, *NRAS*, *BRAF* and *PIK3CA* (Wild-Type, mutated, missing for each) did not meet the 10% change in coefficient criteria for confounder selection.
