# Supplementary material for: Fusobacterium nucleatum tumor DNA levels are associated with survival in colorectal cancer patients
Source: Eur J Clin Microbiol Infect Dis. 2019 Jul 31;38(10):1891–9. doi: 10.1007/s10096-019-03649-1 (PMC6778531; doi:10.1007/s10096-019-03649-1)
Supplement: Supplementary file 2 — (DOCX 16 kb) [file 10096_2019_3649_MOESM2_ESM.docx]

**Supplementary Table 2. Sensitivity analyses using Cox Proportional Hazards models for the association between *Fusobacterium nucleatum* DNA status in tertile categories and overall survival in multivariate analyses.**

| Analysis / Categories^1^ | Person-years | No. of events | Hazard Ratio  (95% confidence Intervals) |
| --- | --- | --- | --- |
| *F. nucleatum* DNA in colorectal cancer tissue (2-∆CT) ^2^ |  |  |  |
| *No or low** | 314.1 | 41 | 1.00 (referent) |
| *Moderate* | 117.4 | 12 | 0.77 (0.40-1.50) |
| *High* | 104.7 | 18 | 1.35 (0.75-2.43) |
| *F. nucleatum* quantification in disease tissue over matched normal colorectal tissue (2−∆∆CT) ^3^ |  |  |  |
| *No or low* | 292.3 | 42 | 1.00 (referent) |
| *Moderate* | 121.6 | 12 | 0.68 (0.34-1.35) |
| *High* | 110.20 | 16 | 1.42 (0.57-2.60) |

All models adjusted for age (<60, 60-<70, 70-<80, 80+), sex (men, women), tumor stage (I, II, III, IV, missing), and chemotherapy or radiotherapy within 6 months (no, yes).

^1^ Categories of no/low refer to values where *F. nucleatum* was not detectable or if the abundance/quantification was in the lowest tertile category, moderate if *F. nucleatum* abundance/quantification was in the middle tertile category, or high if *F. nucleatum* abundance/quantification was in the highest tertile category.

^2^ *Fusobacterium nucleatum* (*F. nucleatum*) levels are given as relative quantification and were determined by 2−∆CT, where ∆CT is the difference in the cycle threshold (CT) number for *F. nucleatum* and the *PGT* reference gene assay.

^3^ *F. nucleatum* quantification was determined as bacterial quantification in disease tissue over adjacent matched mucosal colorectal tissue (2−∆∆CT).
